# Histological review of skin cancers in African Albinos: a 10-year retrospective review

**DOI:** 10.1186/1471-2407-14-157

**Published:** 2014-03-06

**Authors:** Samson Kimaiyo Kiprono, Baraka Michael Chaula, Helmut Beltraminelli

**Affiliations:** 1 Department of Dermatology, Regional Dermatology Training Center, P.O. Box 8332, Moshi, Tanzania; 2 Department of Dermatology, Provincial General Hospital, P.O. Box 15-50100, Kakamega, Kenya; 3 Department of Dermatology, Inselspital - University Hospital, Bern CH-3010, Switzerland

**Keywords:** Albinos, African, Skin Cancer

## Abstract

**Background:**

Skin cancer is rare among Africans and albinism is an established risk for skin cancer in this population. Ultraviolet radiation is highest at the equator and African albinos living close to the equator have the highest risk of developing skin cancers.

**Methods:**

This was a retrospective study that involved histological review of all specimens with skin cancers from African albinos submitted to The Regional Dermatology Training Center in Moshi, Tanzania from 2002 to 2011.

**Results:**

A total of 134 biopsies from 86 patients with a male to female ratio of 1:1 were reviewed. Head and neck was the commonest (n = 75, 56.0%) site affected by skin cancers. Squamous cell carcinoma (SCC) was more common than basal cell carcinoma (BCC) with a ratio of 1.2:1. Only one Acral lentiginous melanoma was reported. Majority (55.6%) of SCC were well differentiated while nodular BCC (75%) was the most common type of BCC.

**Conclusions:**

Squamous cell carcinoma is more common than basal cell carcinoma in African albinos.

## Background

Skin cancer is the most common malignancy among Caucasians. It represents approximately 20-30% of all neoplasms in Caucasians and 1-2% in those with colored skin [1]. Skin cancer is a major cause of morbidity and mortality in Albinos who develop premalignant and malignant lesions at a younger age and suffer from advanced skin cancers in the third to fourth decade of life [2,3].

Albinism is a genetically inherited disorder with a worldwide distribution. Phenotypically it presents with reduced or no melanin in the hair, the skin and the eyes [4]. Genetically albinism is classified into four types according to the type of gene mutation [4]. Oculocutaneous albinism type II (OCAII) is the most common type of albinism in Africa [4]. The prevalence is estimated to range from 1 in 15,000 in the East-Central State of Nigeria [2] to 1 in 1,000 in the Tonga tribe of Zimbabwe [5]. The prevalence in Tanzania is estimated to be 1 in 2,500 inhabitants [3]. The lack of melanin and exposure to intense ultraviolet radiation increase the risk of developing skin cancer. The lack of melanin in Albinos increases the risk of developing skin cancer by 1,000 fold as compared with the general African population [6]. The aim of this study was to determine the common type of skin cancers and if there is an increase in prevalence of squamous cell carcinoma using biopsies and excisions from Albinos.

## Methods

This study was conducted at the Regional Dermatology Training Center (RDTC) at the Kilimanjaro Christian Medical Center. This is a referral hospital for skin diseases in Northern Tanzania with a catchment area population extending to the neighboring countries. This was a retrospective study covering a period of 10 years (from 2002 to 2011). All files of patients who were biopsied or whose tumors had been excised and submitted for histopathological examination were retrieved and a structured data collection tool was used to extract the data. Data was entered into a statistical package for social scientists (SPPSS Chicago Inc.) for descriptive analysis. Ethical clearance was waived by the Kilimanjaro Christian Medical College Ethical Committee. All slides were examined by a dermatopathologist. In case the slides were missing or damaged, new slides were prepared from the stored blocks.

Squamous cell carcinoma (SCC) was classified in three categories according to the most poorly differentiated site within each specimen [7]:

*Well differentiated:* Squamous epithelium with easily recognizable and abundant keratinization. Nuclear and cellular pleomorphism is minimal while mitotic figures are few and basally located.

*Moderately differentiated:* Structural disorganization of squamous epithelium with pronounced pleomorphism and more mitotic figures. Keratinization is limited to keratin pearls.

*Poorly differentiated*: Anaplastic tumor with foci of keratinization or originating from the surface epithelium.

## Results

**Figure 1:**
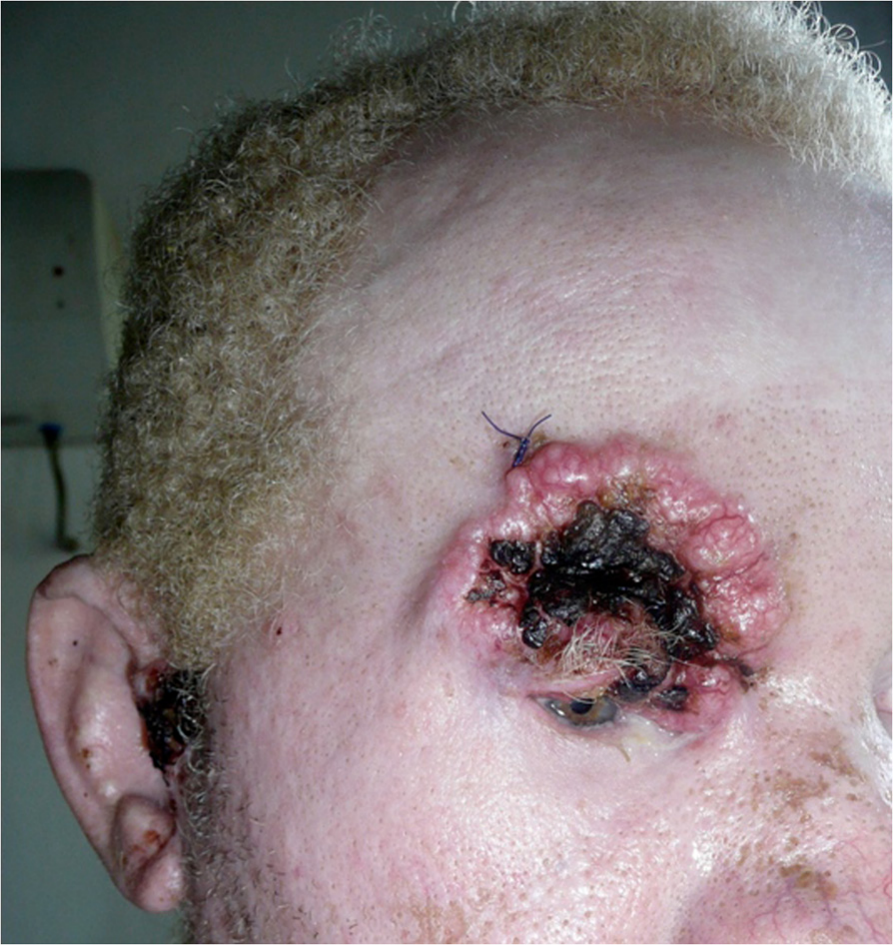
Showing supraorbital ulcerated, nodular basal cell carcinoma and an ulcerated preauricular squamous cell carcinoma in a 23 years old African albino.

**Figure 2:**
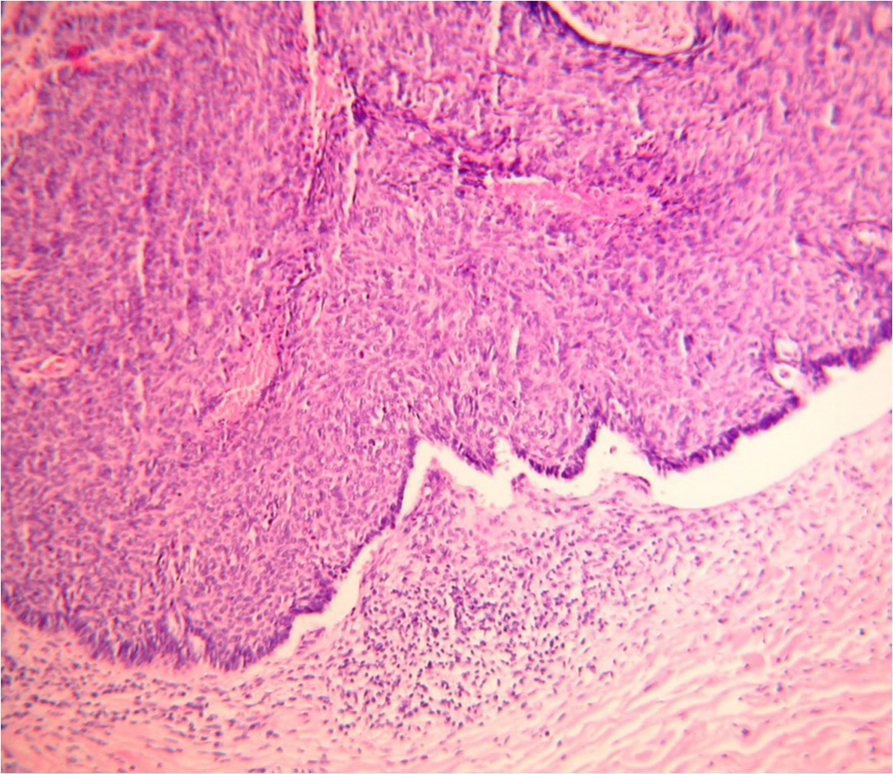
Histological picture showing a section of a nodular basal cell carcinoma (Hematoxylin and eosin, x10).

A total of 134 histological biopsies from 86 patients were reviewed. The biopsies were from almost equal number of each gender with a male to female ratio of 1:1.1. The youngest patient with skin cancer was aged 18 years and the oldest was 68 years (median age was 34 years) with a mean of 35.5 years (SD ±19.5). The duration of skin cancer ranged from 3 months to 120 months with a mean of 14.1 months (SD ± 19.5). However, the duration of skin cancer was not indicated in 28 files. The head and neck was affected in 75 (56.0%) cases while the trunk and the extremities were affected in 40 (30%) and 19 (14%) patients respectively. The tumors were ulcerated in 39 (29%) patients. Some patients presented with multiple ulcerated tumours as shown on Figure 1. The most common (53.7%) tumor was SCC and only one acral lentiginous melanoma was reported. The majority (55.6%) of the SCC were well differentiated, whereas nodular basal cell carcinoma (BCC) shown in Figure 2 was the most predominant (73.8%) type. The ratio of BCC to SCC was 1:1.2 as shown in Table 1. The specimen of melanoma represented a small part of a large tumour and therefore the Clark level and Breslow thickness could not be determined.

**Table 1:** Characteristics of 134 skin cancers from biopsies and excisions in Albinos

	**Frequency**	**Percentage**
**Type of the tumor (n = 134)**		
Squamous cell carcinomas (SCCs)	72	53.7
Basal cell carcinomas (BCCs)	61	45.5
Melanoma	1	0.75
**Type of SCCs**		
Well differentiated	36	50.0
Moderately differentiated	31	43.1
Poorly differentiated	5	6.9
**Type of BCCs**		
Nodular BCCs	45	73.8
Superficial BCCs	7	11.5
Infiltrating BCCs	9	14.8

## Discussion

This study is the largest histological review of skin cancers in African albinos. Previous studies reported results from small sample size with the largest study involving 64 cases (8). Albinos living close to the equator develop sun damage earlier in life because of lack of sun protection and they develop premalignant and malignant tumors by the second decade of life. Previous studies from Nigeria [2] and Tanzania [3] reported that only a few Albinos survived beyond 30 years. However, the findings from this study showed a mean age of 35 years (SD ± 10.6) representing an increase in the survival rate, which could be due to improved awareness and early management of skin cancer in general, and in particular this could be a positive consequence of the ongoing Albino outreach program in the RDTC since 1993.

All the tumors were non- melanoma skin cancers (NMSCs) except for one case of melanoma. This overwhelming predominance of NMSCs over melanoma has also been observed in Caucasians. The incidence of NMSCs is reported to be 18 - 20 times that of malignant melanoma [8]. Non- melanoma skin cancers mainly affect older individuals, while the frequency of melanoma peaks at 20-45 years [8]. The age of the majority of the patients in this study was within the peak age for melanoma, but much lower for NMSCs. This observation indicates that melanoma is rare in Albinos, a finding that has also been reported in other studies [9,10]. The relationship between the exposure to ultraviolet radiation and the risk for developing melanoma is not clear-cut and the incidence rates relate to the latitude to a lesser extent than that for NMSCs [11]. The complex association between the exposure to ultraviolet radiation and other factors including genetic factors may explain the low melanoma incidence.

The majority of NMSCs in Caucasians are BCCs and SCCs. The incidence of BCCs is estimated to be 4 times higher than that for SCCs [12]. In contrast to this study, there were more SCCs than BCCs with BCCs to SCCs ratio of 1:1.2. Previous studies have reported the predominance of SCCs in Albinos with SCCs occurring 3 - 6 times more than BCCs [9,10,13-15]. The relatively high predominance of SCCs over BCCs in those studies could have been due to the small sample sizes in which four studies had less than 20 patients. Those studies were also based on excisional biopsies from advanced tumors, whereas biopsies in this study were obtained from patients undergoing routine dermatological examination and advanced tumors. The slowly growing small BCC may have been missed in previous studies. High cumulative dose of ultraviolet radiation is associated with the development of SCCs. The incidence of SCCs doubles for every 8-10 degrees decline in the latitude with the maximum incidence at the equator [8]. The ultraviolet dose per unit time at the equator is about 200% of that in Europe or Northern USA [16].

Skin cancers generally develop at body sites exposed to ultraviolet radiation [9,10,12]. A significant number (40) of skin tumors occurred on the trunk. The hot weather conditions and the farming activities in this community limit the use of proper sun protective clothing, which may increase the incidence of skin cancers on the otherwise sun protected areas.

The mean duration of skin cancer before seeking medical care of 14.4 months was almost similar to the 17.9 months reported by Alexander and Henschke [15] 30 years ago. The delay in seeking medical treatment for skin cancer of a mean of 26 months was also reported in Nigeria [13]. Albinos also face social discrimination [17] and have difficulties in formal education because of poor eyesight [5]. Poverty, illiteracy and social discrimination contribute in the delay to seek medical care especially for skin cancer. This study was based on biopsies or tumor excisions that underwent histopathological examination. However, tumors that were excised, but not submitted for histopathological examination or not biopsied were missed. Therefore, this study may be biased.

## Conclusions

Non-melanoma skin cancers are overwhelmingly predominant in Albinos. The proportion of SCCs and BCCs is almost identical. These occur at a younger age, but there is a considerable patient delay in seeking medical care.

## Consent

Written informed consent was obtained from the patient for publication of this image.

## Competing interests

All authors declare no competing interest.

## Authors’ contributions

SK and BC conceived and participated in the design of the study. HB analyst histological slides. SK drafted the manuscript. All Authors read and approved the final manuscript.

## Pre-publication history

The pre-publication history for this paper can be accessed here:

http://www.biomedcentral.com/1471-2407/14/157/prepub
